# GBDTLRL2D Predicts LncRNA–Disease Associations Using MetaGraph2Vec and K-Means Based on Heterogeneous Network

**DOI:** 10.3389/fcell.2021.753027

**Published:** 2021-12-17

**Authors:** Tao Duan, Zhufang Kuang, Jiaqi Wang, Zhihao Ma

**Affiliations:** School of Computer and Information Engineering, Central South University of Forestry and Technology, Changsha, China

**Keywords:** long noncoding RNA, heterogeneous network, MetaGraph2Vec, K-means, Gradient Boosting Decision Tree, logistic regression

## Abstract

In recent years, the long noncoding RNA (lncRNA) has been shown to be involved in many disease processes. The prediction of the lncRNA–disease association is helpful to clarify the mechanism of disease occurrence and bring some new methods of disease prevention and treatment. The current methods for predicting the potential lncRNA–disease association seldom consider the heterogeneous networks with complex node paths, and these methods have the problem of unbalanced positive and negative samples. To solve this problem, a method based on the Gradient Boosting Decision Tree (GBDT) and logistic regression (LR) to predict the lncRNA–disease association (GBDTLRL2D) is proposed in this paper. MetaGraph2Vec is used for feature learning, and negative sample sets are selected by using K-means clustering. The innovation of the GBDTLRL2D is that the clustering algorithm is used to select a representative negative sample set, and the use of MetaGraph2Vec can better retain the semantic and structural features in heterogeneous networks. The average area under the receiver operating characteristic curve (AUC) values of GBDTLRL2D obtained on the three datasets are 0.98, 0.98, and 0.96 in 10-fold cross-validation.

## 1 Introduction

In the human genome, more than 98*%* of the genes are noncoding protein sequences. The remaining 2*%* can only be transcribed into noncoding RNAs (ncRNAs). The ncRNAs can be divided into microRNA (miRNA), long ncRNA (lncRNA), etc. NcRNAs between 200 and 100,000 in length are lncRNAs.

At first, lncRNAs are considered useless RNAs without any biological function (Mercer and Mattick, 2013). This is because they are expressed at a lower level than protein-coding RNAs. However, with the development of experimental methods and computing power, the change of lncRNA has been found to be associated with many diseases, such as colorectal cancer (Xiong et al., 2021), lung adenocarcinoma (Hou et al., 2021), and gastrointestinal cancer (Abdi et al., 2021). With the deepening of research on lncRNAs, there are many pieces of evidence that lncRNAs play a key role in many important biological processes, including transcription, translation, splicing, differentiation, epigenetic regulation, immune response, and cell cycle control. For example, lncRNA loc105377478 promotes NPs-Nd2O3 in 16HBE cells and thus induces inflammation in human bronchial epithelial cells (Yu et al., 2021). LncRNA HOTAIR is considered a potential biomarker (Maass et al., 2014). The expression level of HOTAIR in breast cancer tissues is 100 to approximately 2,000 times higher than normal tissues and is associated with the proliferation and survival of colorectal cancer (Ge et al., 2013). Some research has shown that lncRNA BCAR4 is expressed in 27*%* of primary breast tumors. LncRNA Braveheart has also been demonstrated to control heart development by interacting with the epigenetic modifier PRC2. And increasing the expression of lncRNA Linc-MD1 can promote muscle differentiation (Cesana et al., 2011). LncRNA NEAT1 can regulate the development of Parkinson's. Therefore, the research of the potential lncRNA–disease association can better comprehend the potential mechanism of human diseases and help diagnose and treat diseases. This research has important practical implications.

Biological experiments to identify potential associations are time-consuming, labor-intensive, and very expensive. Therefore, in order to effectively reduce the time consumed by biological experiments and economic costs, there has been much research based on bioinformatics and computational power. For example, the method KATZLGO is proposed by Zhang et al. (2017) to predict the interaction of lncRNA–lncRNA. The PLPIHS is proposed by Xiao et al. (2017) to predict lncRNA–protein interactions using HeteSim score. A computational framework for predicting lncRNA–protein interactions is proposed by Liu (2020). An approach to explore miRNA sponge networks in breast cancer is proposed by Tian and Wang (2021). A method to predict the subcellular localization of lncRNAs is proposed by Yang et al. (2020). The potential roles of oral squamous cell carcinoma (OSCC)-related mRNA and lncRNA are revealed by Li et al. (2021) through protein interaction network and co-expression network analysis. The model GBDTL2E is proposed by Wang et al. (2020) to predict the association between lncRNA and environmental factors. With the deepening of research, research on the prediction of lncRNA–disease association is mainly divided into the following categories:

1) Based on machine learning methods, the main idea of these methods is to prioritize candidate lncRNAs by training known and unknown lncRNA–disease correlation. The semi-supervised learning framework LRLSLDA is proposed by Chen and Yan (2013). A graph regularization non-negative matrix factorization (LDGRNMF) is proposed by Wang M.-N. et al. (2021). Based on the weight algorithm and the improved projection algorithm, LDAP-WMPS is proposed by Wang B. et al. (2021). A model proposed by Zhou et al. (2021) uses high-order proximity reserved embedding to embed nodes into the network. The model VGAELDA, which integrates variational reasoning and graph autoencoder, is proposed by Shi et al. (2021). A multi-label fusion collaborative matrix decomposition (MLFCMF) method is proposed by Gao et al. (2021) to predict lncRNA–disease associations. The model PSPA-LA-PCRA is proposed by Wang and Zhang (2021), which uses the data of pathological stages. The random distribution logical regression framework (RDLRF) is proposed by Sun et al. (2021), and the RDLRF combines simboost feature extraction with logistic regression (LR). The method FVTLDA is proposed by Xiao et al. (2020), which combines multiple linear regression and artificial neural network. The BLM-NPA is proposed by Cui et al. (2019) to predict based on the nearest neighbor. The alternate least squares method of matrix factor factorization (ALSBMF) is proposed by Zhu et al. (2020). A computational method based on graphical autoencoder matrix completion (GAMCLDA) is proposed by Wu et al. (2020). A deep matrix factorization method (DMFLDA) is proposed by Zeng et al. (2020). A graph-based method (PANDA) is proposed by Silva and Spinosa (2021). The PANDA takes the association prediction of lncRNAs and diseases as a link prediction problem.

2) Based on network methods, the main idea of these methods is using a similarity network to predict lncRNA–disease association. Based on the combination of incremental principal component analysis (IPCA) and random forest (RF), a lncRNA–disease association prediction method IPCARF is proposed by Zhu et al. (2021). The prediction method for lncRNA–disease associations (PCSLDA) based on Point Cut Set is proposed by Kuang et al. (2019). The model GAERF is proposed by Wu et al. (2021); GAREF uses graph autocoding (GAE) and RF to identify disease-related lncRNAs. A random walk-based multi-similarity fusion and bidirectional label propagation method RWSF-BLP is proposed by Xie et al. (2021). Based on the assumption that there is a potential association between an lncRNA and a disease, if they are associated with the same set of miRNAs, similar diseases tend to be closely related to IneRNAs with similar functions; the method LDLMD is proposed by (Wang et al., 2019). A method of internal confidence-based local radial basis biological network (ICLRBBN) is proposed by Wang Y. et al. (2021). A two-stage prediction model (DRW-BNSP) is proposed by Zhang et al. (2021). HAUBRW algorithm is proposed by Xie et al. (2020b), which combines thermal diffusion algorithm and probabilistic diffusion algorithm to redistribute resources. An lncRNA–disease association prediction model based on RF and feature selection, RFLDA, is proposed by Yao et al. (2020). A predictive lncRNA–disease prediction model based on heterogeneous networks is proposed by Song et al. (2020). The LDAMAN is proposed by Zhang et al. (2020), which uses a structural deep network embedding model. The method based on linear neighborhood similarity and unbalanced double random walk (LDA-LNSUBRW) is proposed by Xie et al. (2020a). The MHRWR is proposed by Zhao et al. (2020) to integrate the similarity network of lncRNAs, diseases, and genes, with the known lncRNA–disease association network, lncRNA–gene network, and disease–gene network. The method LDAH2V is proposed by Deng et al. (2021), which uses HIN2Vec to calculate the meta path and eigenvector of each lncRNA–disease pair in heterogeneous information networks.

It can be seen that the association prediction of lncRNAs and diseases has become a research hotspot. Currently, the existing methods simply regard all objects in the network as the same type. However, in heterogeneous networks, there are many types of nodes, and the relationship between nodes is very complex, which is not considered by traditional methods. At the same time, unknown correlation is far greater than known correlation, which brings great challenges to model training. To solve these problems, a method based on the Gradient Boosting Decision Tree (GBDT) and LR to predict the lncRNA–disease association (GBDTLRL2D) is proposed in this paper. The GBDTLRL2D uses MetaGraph2Vec for feature learning and the K-means clustering method to select negative sample sets. The contributions of our method are included:

• The GBDTLRL2D comprehensively considers the topological structure characteristics and meta-path characteristics of nodes in heterogeneous networks. MetaGraph2Vec is used to learn more information by capturing more semantic relationships between remote nodes.
• The GBDTLRL2D uses the K-means to get the clustering of the unknown correlation. The same number of negative samples as the positive samples is selected from the clusters.
• The GBDTLRL2D combines GBDT and LR. The GBDT + LR is a special classification algorithm. Its ability to find features and combine features is very powerful. The classification accuracy is high.

## 2 Materials and Methods

The known lncRNA and disease-associated data used in this paper are downloaded from the lncRNADisease (Chen et al., 2012), which includes three versions, namely, the version of June 2012, the version of January 2014, and the version of June 2015. Table 1 shows the data after deduplication.

**TABLE 1:** LncRNA–disease association relationship dataset.

Dataset	Number of lncRNA	Number of diseases	Number of associations
DataSet1 (DS1)	112	150	276
DataSet2 (DS2)	131	169	319
DataSet3 (DS3)	285	226	621

Note. lncRNA, long noncoding RNA.

**FIGURE 1:**
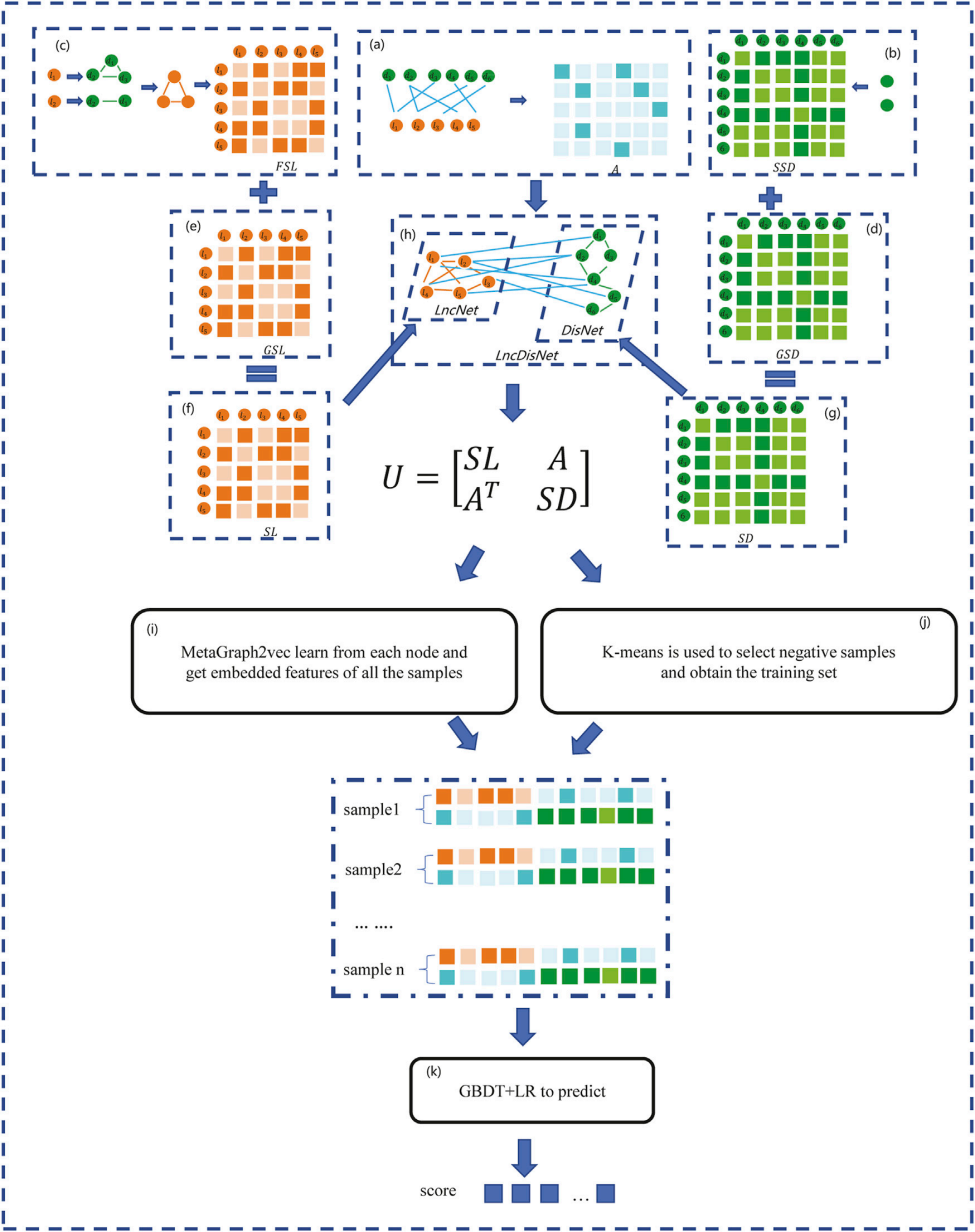
Flowchart of the GBDTLRL2D. **(A)** Obtained the association matrix A. **(B)** Calculated the disease semantic similarity matrix SSD. **(C)** Calculated the long noncoding RNA (lncRNA) functional similarity matrix FSL. **(D)** Calculated the disease Gaussian interaction profile kernel similarity GSD. **(E)** Calculated the lncRNA Gaussian interaction profile kernel similarity GSL. **(F)** Obtained the lncRNA similarity SL. **(G)** Obtained the disease similarity SD. **(H)** Integrated three subnets A, SL, and SD to construct a global heterogeneous network. **(I)** Obtained the embedded features of nodes. **(J)** Selected the negative sample and obtained the training set. **(K)** Trained the Gradient Boosting Decision Tree combined with logistic regression classifier (GBDT + LR).

In this section, a method based on the GBDT and LR to predict the lncRNA–disease association (GBDTLRL2D) is proposed. The GBDTLRL2D uses MetaGraph2Vec for feature learning and the K-means clustering method to select negative sample sets. The main steps of GBDTLRL2D are as follows: 1) according to the downloaded data, the set of lncRNAs and diseases as well as the association matrix A of lncRNA–diseases is obtained after deduplication. 2) The disease semantic similarity matrix SSD and lncRNA functional similarity matrix FSL are calculated, and then the Gaussian interaction profile kernel similarity matrix of disease (GSD) and lncRNA (GSL) are calculated. 3) LncRNA similarity matrix SL is constructed according to GSL and FSL, and disease similarity matrix SD is constructed according to GSD and SSD. 4) The association matrix A of lncRNA–disease, lncRNA similarity matrix SL, and the disease similarity matrix SD are integrated to construct the global heterogeneous network G. On the G, the feature of each node is learned by MetaGraph2Vec to obtain the feature representation of each node. 5) K-means is used to select negative samples to obtain train sets. 6) The GBDT and LR classifier are used to predict the lncRNA–disease association. Figure 1 is a flowchart of the GBDTLRL2D. Each step of GBDTLRL2D is detailed in the next section.

### 2.1 Calculate Disease Semantic Similarity

**FIGURE 2:**
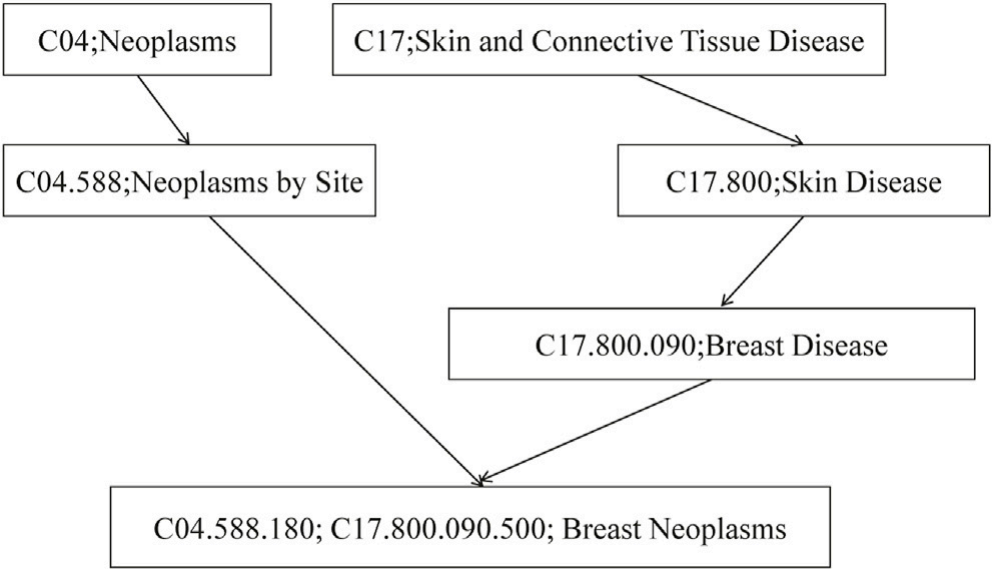
The disease DAG of breast neoplasms. DAG, directed acyclic graph.

**FIGURE 3:**
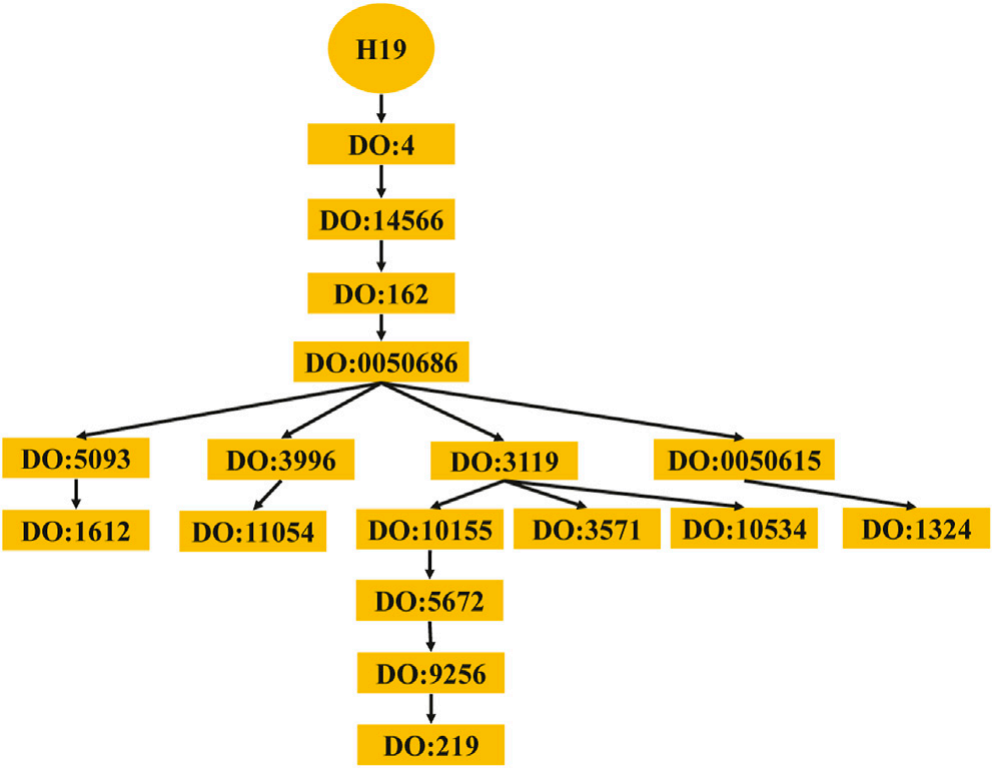
Diseases associated with lncRNA H19. lncRNA, long noncoding RNA.

The computing method of disease semantic similarity SSD in this experiment is based on the Disease Ontology. The method presents the disease tissue as a directed acyclic graph (DAG). As shown in Figure 2, the relationships between diseases are described in a DAG. Each node is a disease, and the arrow points from a disease to its ancestor disease. It can be seen from Figure 3 that if one lncRNA is associated with a disease, then this lncRNA may be associated with sub-diseases of the disease. From this perspective, the correct identification of these new associations may help to understand the mechanisms underlying RNA levels and improve the speed of accurate diagnosis and treatment of diseases. Therefore, the SSD between diseases is calculated according to the DAG.

For disease *d*_*i*_, the semantic value is obtained. The contribution of each ancestral disease in the DAG of disease *d*_*u*_ to the semantic value of *d*_*i*_ is firstly calculated as shown in formula (1):

$$C_{i}\left(u\right)=\left\{\begin{array}{cc} 1,\; & \text{ if }u=i\\ \max\left\{\Delta \times C_{i}\left(u^{\prime }\right)|u^{\prime }\in \text{ children of }u\right\},\; & \text{ if }u\neq i \end{array}\right.$$ (1)

where Δ is the weight of the edge connecting disease *d*_*u*_ and its sub-diseases, namely, semantic contribution factor. According to the above formula, as the distance between disease *d*_*i*_ and other diseases increases, semantic contributions decrease. Therefore, Δ should be selected between 0 and 1. In this paper, Δ= 0.5. Then the semantic value of *d*_*i*_ is calculated as the sum of the contributions of *d*_*i*_’s ancestor disease and *d*_*i*_ itself, as shown in formula (2):

$$C\left(i\right)=\underset{u\in Z\left(i\right)}{\sum }C_{i}\left(u\right)$$ (2)

where Z(*i*) represents the node set in the DAG of disease *d*_*i*_.

For the disease *d*_*i*_ and *d*_*j*_, when the DAG of disease *d*_*i*_ and *d*_*j*_ has more overlapping nodes, their semantic similarity is higher. Therefore, the semantic similarity matrix SSD of diseases can be obtained as shown in formula (3):

$$\mathrm{SSD}\left(i,j\right)=\frac{\sum_{u\in Z\left(i\right)\cap Z\left(j\right)}\left(C_{i}\left(u\right)+C_{j}\left(u\right)\right)}{C\left(i\right)+C\left(j\right)}$$ (3)

SSD (*i*, *j*) is denoted as the semantic similarity value between disease *d*_*i*_ and *d*_*j*_.

### 2.2 Calculate Long Noncoding RNA Functional Similarity

According to the LNCSIM (Chen et al., 2015), the lncRNA functional similarity is described as follows. Diseases associated with the same lncRNA are grouped into a set. The DL1 is the disease set related to lncRNA *l*_*m*_, including *x* diseases. The DL2 is the disease set related to lncRNA *l*_*n*_, including *y* diseases. When the semantic similarity between diseases in DS1 and DS2 is higher, the functional similarity between lncRNA *l*_*m*_ and *l*_*n*_ may be higher, as shown in formula (4):

$$\mathrm{FSL}\left(l_{m},l_{n}\right)=\frac{\sum_{d\in \mathrm{DL}2}maxS\left(d,\mathrm{DL}1\right)+\sum_{d\in \mathrm{DL}1}maxS\left(d,\mathrm{DL}2\right)}{x+y}$$ (4)

$$\mathrm{maxS}\left(d,\mathrm{DL}1\right)=\max_{d\in DL1}\left(\mathrm{SS}\left(d,d_{1}\right)\right)$$ (5)

where maxS (*d*, DL1 (*l*_*m*_)) is the maximum semantic similarity of all diseases in the set DL1 related to lncRNA *l*_*m*_.

### 2.3 Calculate Gaussian Interaction Profile Kernel Similarity

In this section, the adjacency matrix A is constructed according to the known lncRNA–disease association. The A (*l*_*i*_, *d*_*j*_) indicates whether lncRNA *l*_*i*_ and disease *d*_*j*_ are related. The A as shown in formula (6):

$$A\left(l_{i},d_{j}\right)=\left\{\begin{array}{cc} 1\; & l_{i}\text{ is associated with }d_{j}\\ 0\; & \text{ otherwise } \end{array}\right.$$ (6)

In lncRNA functional similarity matrix FSL and the disease semantic similarity matrix SSD, many elements are 0. In order to make the similarity network not sparse, Gaussian kernel similarity is calculated, as shown in formula (7):

$$\mathrm{GSL}\left(l_{m},l_{n}\right)=\exp\left(-\delta_{l}\|A\left(m,:\right)-A\left(n,:\right){\|}^{2}\right)$$ (7)

$$\mathrm{GSD}\left(d_{i},d_{j}\right)=\exp\left(-\delta_{d}{\left\|A\left(:,i\right)-A\left(:,j\right)\right\|}^{2}\right)$$ (8)

where GSL (*l*_*m*_, *l*_*n*_) is the Gaussian interaction profile kernel similarity score of lncRNA *l*_*m*_ and *l*_*n*_, and GSD (*d*_*i*_, *d*_*j*_) is the Gaussian interaction profile kernel similarity of diseases *d*_*i*_ and *d*_*j*_. The A (*m*,) is *m*th row of A, and A (. *i*) is *i*th col of A. Parameters *δ*_*l*_ and *δ*_*d*_ are obtained as shown in Eq. 9 and Eq. 10:

$$\delta_{l}=\delta_{l}^{\prime }/\left(\frac{1}{p}\overset{p}{\underset{m=1}{\sum }}{\left\|A\left(m,:\right)\right\|}^{2}\right)$$ (9)

$$\delta_{d}=\delta_{d}^{\prime }/\left(\frac{1}{q}\overset{q}{\underset{i=1}{\sum }}{\left\|A\left(:,i\right)\right\|}^{2}\right)$$ (10)

where *p* is the number of lncRNAs and *q* is the number of diseases.

### 2.4 Obtain Similarity Network

In this section, lncRNA similarity network and disease similarity network are constructed. The lncRNA similarity network is represented as SL. SL is fused by FSL and GSL. For lncRNA *l*_*m*_ and *l*_*n*_, if FSL (*l*_*m*_, *l*_*n*_) = 0, then SL (*l*_*m*_, *l*_*n*_) = GSL (*l*_*m*_, *l*_*n*_); otherwise, SL (*l*_*m*_, *l*_*n*_) = FSL (*l*_*m*_, *l*_*n*_), as shown in formula (11):

$$\mathrm{SL}\left(l_{m},l_{n}\right)=\left\{\begin{array}{cc} \mathrm{GSL}\left(l_{m},l_{n}\right)\; & \text{ if }\mathrm{FSL}\left(l_{m},l_{n}\right)=0\\ \mathrm{FSL}\left(l_{m},l_{n}\right)\; & \text{ otherwise } \end{array}\right.$$ (11)

$$\mathrm{SD}\left(d_{i},d_{j}\right)=\left\{\begin{array}{cc} \mathrm{GSD}\left(d_{i},d_{j}\right)\; & \text{ if }\mathrm{SSD}\left(d_{i},d_{j}\right)=0\\ \mathrm{SSD}\left(d_{i},d_{j}\right)\; & \text{ otherwise } \end{array}\right.$$ (12)

Similarly, the disease similarity network is expressed as SD. The SD is fused by SSD and GSD, as shown in formula (12):

### 2.5 Obtain Node Features Through MetaGraph2Vec

In this section, the heterogeneous network is constructed by integrating the adjacency matrix A of lncRNA–disease association, lncRNA similarity network SL, and the disease similarity network SD. Because there are many types of nodes and complex relationships among nodes in the heterogeneous network, embedding networks is difficult to implement. In GBDTLRL2D algorithm, MetaGraph2Vec (Zhang et al., 2018) is used for feature learning, which preserves both structural and semantic features in heterogeneous networks. MetaGraph2Vec uses metagraph to guide the generation of a random walk and learn about the potential embedding of nodes in a heterogeneous network of multiple types. Metagraph can represent features of sparse heterogeneous networks based on meta-paths. The specific substeps are shown below.

#### 2.5.1 Building Heterogeneous Networks

The heterogeneous network G = (*V*, *E*) is constructed, where *V* represents the set of nodes and *E* represents the set of edges. The G is denoted as U. The dimension of the matrix U is (*nl* + *nd*)∗(*nl* + *nd*), where *nl* is the number of lncRNAs and *nd* is the number of diseases, as shown in formula (13):

$$U=\left[\begin{array}{cc} \mathrm{SL} & A\\ A^{T} & \mathrm{SD} \end{array}\right]$$ (13)

where *A* ^T^ is the transpose of A.

#### 2.5.2 Random Walk Guided by metagraph

Given a metaGraph *g* = (*N*, *M*, *n*_*s*_, *n*_*t*_) with *n*_*s*_ = *n*_*t*_, the metaGraph is defined as a DAG on G, where *n*_*s*_ represents the source node, *n*_*t*_ represents the target node, *N* is the node set, and *M* is the edge set. The $g^{\infty }=\left(N,M,n_{s}^{\infty },n_{t}^{\infty }\right)$ is recursive metaGraph of *g*. The *g* ^*∞*^ is constructed by any number of *g*’s connected tail to head. A node of type *n*_*s*_ is selected to start a random walk guided by the metatype.

In step *i*, node *v*_*i*−1_ is selected as the start of a random walk guided by the metaGraph. The random walk obtains the edge types of node *v*_*i*−1_ in a heterogeneous network that meets the constraints in the metagraph with all neighboring nodes. One edge type is randomly selected. Then, an edge of the selected edge type is randomly selected to get the next node *v*_*i*_. The random walk terminates when there is no edge type that satisfies the constraint.

The transition probability of step *i* guided by metaGraph *g* is denoted as T (*v*_*i*_∣*v*_*i*−1_; *g* ^*∞*^), *v*_*i*−1_ is the current node, and *v*_*i*_ is the next hop node. If the node *v*_*i*−1_ and its neighbors in the heterogeneous network G do not satisfy the edge type of the constraint of the *g* ^*∞*^, T (*v*_*i*_∣*v*_*i*−1_; *g* ^*∞*^) = 0. Otherwise, T (*v*_*i*_∣*v*_*i*−1_; *g* ^*∞*^) is shown in formula (14):

$$T\left(v_{i}|v_{i-1};g^{\infty }\right)=\frac{1}{NUM_{g^{\infty }}\left(v_{i-1}\right)}\times \frac{1}{\left|\left\{\mu |\left(v_{i-1},\mu \right)\in E,\phi \left(v_{i}\right)=\phi \left(\mu \right)\right\}\right|}$$ (14)

where $\left|\left\{\mu |\left(v_{i-1},\mu \right)\in E,\phi \left(v_{i}\right)=\phi \left(\mu \right)\right\}\right|$ is the number of neighbor nodes of the same type as *v*_*i*−1_. $NUM_{g^{\infty }}\left(v_{i-1}\right)$ is the number of edge types that satisfy the constraint in the *g* ^*∞*^ starting from *v*_*i*−1_, as shown in formula (15):

$$NUM_{g^{\infty }}\left(v_{i-1}\right)=\left|\left\{j\left|\left(\phi \left(v_{\text{i}-1}\right),\phi \left(\mu \right)\right)\cdot \in M\cap \left(N\left[l^{\infty }\left(\phi \left(v_{\text{i}-1}\right)\right)\right]\times N\left[j\right]\right),\right|\left(v_{\text{i}-1},\mu \right)\in E\right\}\right|$$ (15)

After several walks, a node sequence *S*_*g*_ = *v*_1_, *v*_2_, …, *v*_*L*_ of length L is finally obtained.

#### 2.5.3 Obtain Node Features Through MetaGraph2Vec

By learning the mapping function Ψ, the nodes of heterogeneous networks are embedded into a *d*-dimensional space to obtain the embedding feature. The network G has a large number of nodes with different semantics. The nodes with similar semantics in heterogeneous networks are guaranteed to have similar low-dimensional representations Ψ(*v*).

The node sequence *S*_*g*_ = *v*_1_, *v*_2_, …, *v*_*L*_ of length L is obtained by a random walk guided by metaGraph *g*. The embedding function Ψ(⋅) is learned by maximizing the occurrence probability of nodes before and after *v*_*i*_ in the window, and the window size is *b*. Ψ(⋅) is shown in formula (16):

$$\min_{\Psi }-\log T\left(\left\{v_{i-b},\ldots ,v_{i+b}\right\}\backslash /v_{i}|\Psi \left(v_{i}\right)\right)$$ (16)

where $T\left(\left\{v_{i-b},\ldots ,v_{i+b}\right\}\backslash /v_{i}|\Psi \left(v_{i}\right)\right)=\prod_{j=i-b,j\neq i}^{i+b}T\left(v_{j}|\Psi \left(v_{i}\right)\right)$ .

Following MetaPath2Vec, the *T* (*v*_*j*_∣Ψ(*v*_*i*_)) is related to the type of *v*_*j*_, as shown in formula (17):

$$T\left(v_{j}|\Psi \left(v_{i}\right)\right)=T\left(v_{j}|\Psi \left(v_{i}\right),\psi \left(v_{j}\right)\right)T\left(\psi \left(v_{j}\right)|\Psi \left(v_{i}\right)\right)$$ (17)

where the probability $T\left(v_{j}|\Psi \left(v_{i}\right),\psi \left(v_{j}\right)\right)$ is shown in formula (18):

$$T\left(v_{j}|\Psi \left(v_{i}\right),\psi \left(v_{j}\right)\right)=\frac{\exp\left(\Phi \left(v_{j}\right)\cdot \Psi \left(v_{i}\right)\right)}{\sum_{\mu \in V,\psi \left(\mu \right)=\psi \left(v_{j}\right)}\exp\left(\Phi^{\prime }\left(\mu \right)\cdot \Psi \left(v_{i}\right)\right)}$$ (18)

After that, stochastic gradient descent is used to learn the parameters. At each iteration, a node context pair (*v*_*i*_, *v*_*j*_) is sampled according to the distribution of *P* (*v*_*i*_, *v*_*j*_), and the *P* (*v*_*i*_, *v*_*j*_) is the occurrence frequency of each node context pair (*v*_*i*_, *v*_*j*_) within *b* window size. The gradients are updated to minimize the following objective:

$$O_{ij}=-\log T\left(v_{j}|\Psi \left(v_{i}\right)\right)$$ (19)

To speed up training, negative sampling is used to approximate the objective function:

$$O_{ij}=\log\rho \left(\Phi \left(v_{j}\right)\cdot \Psi \left(v_{i}\right)\right)+\overset{U}{\underset{u=1}{\sum }}\log\rho \left(-\Phi \left(v_{N_{j,u}}\right)\cdot \Psi \left(v_{i}\right)\right)$$ (20)

where *ρ*(⋅) is the sigmoid function, $v_{N_{j,u}}$ is the *u*th negative node sampled for node *v*_*j*_, and *U* is the number of negative samples, $v_{N_{j,u}}$ sampled from nodes with type Ψ(*v*_*j*_). Formally, parameters Ψ and Φ are updated as follows:

$$\Psi =\Psi -\lambda \frac{\partial O_{ij}}{\partial \Psi };\Phi =\Phi -\lambda \frac{\partial O_{ij}}{\partial \Phi }$$ (21)

where *λ* is the learning rate.

The embedding function Ψ embeds the nodes of a heterogeneous network into a low-dimensional space, embedding each node and obtaining a low-dimensional representation Φ(*v*). Finally, the *d*-dimensional matrix X is obtained.

### 2.6 Obtain Negative Samples Using K-Means Clustering

Since the number of negative samples is far greater than that of positive samples in the set, it is necessary to balance the train set. The proposed method uses a novel and advanced data balancing method K-means clustering. The K-means is a segmentation technique based on centroid. The centroid of a cluster is used to represent the cluster. The centroid of a cluster is defined as the mean value of points within the cluster. The K-means is relatively simple and easy to implement. The specific implementation steps are as follows:

1. The initial k cluster centers are randomly selected from the unknown sample.
2. According to the distance from the point to the center of each cluster, the point is assigned to the closest cluster center category.
3. All points are assigned, and k cluster centers are recalculated.
4. If the recalculated k cluster centers are not the same as the previous cluster centers, we go to 2; otherwise, go to 5.
5. The clustering center is not changed, and the clustering results are output.

**FIGURE 4:**
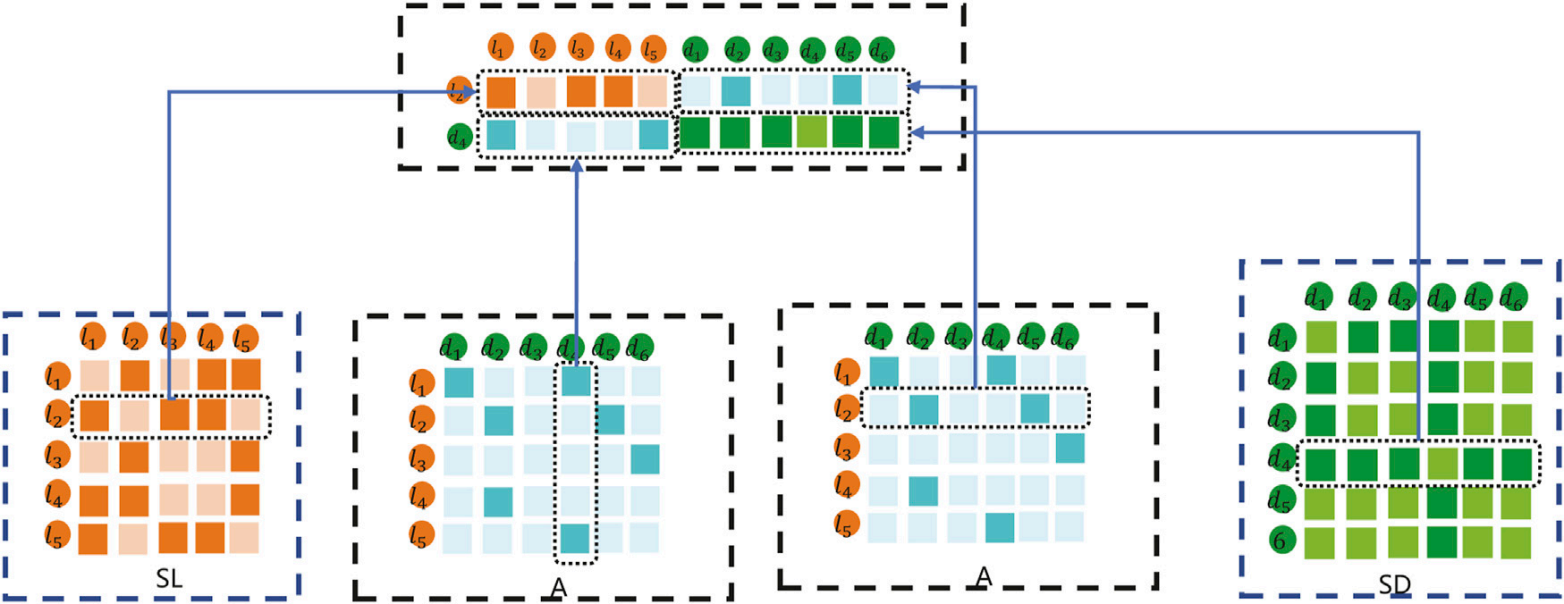
An example of K-means embedding matrix.

In this method, the data feature input into the K-means clustering method is composed of the fusion of SL, SD, and A. For example, the embedding matrix for sample lncRNA *l*_2_ and disease *d*_4_ pairs is shown in Figure 4.

As shown in Figure 4, the embedding matrix of lncRNA *l*_2_ and disease *d*_4_ pairs includes the following parts: 1) the first part is the second row of lncRNA similarity matrix SL; 2) the second part is composed of the vector corresponding to the adjacency matrix A of *d*_4_; 3) the third part is composed of the vector corresponding to *l*_2_ of the adjacency matrix A; and 4) the fourth part is the second row of disease similarity matrix SD. Combined with the representations in the first part, second part, third part, and fourth part, the final lncRNA *l*_2_ and disease *d*_4_ samples are constructed to carry out the K-means embedding matrix.

### 2.7 Train the Gradient Boosting Decision Tree Combined With Logistic Regression Classifier

After the sample and features are obtained, the GBDT + LR classifier is trained. Parameters of the model are initialized. The training data are regressed through the GBDT model and generate a decision tree. The leaf nodes of the decision tree are combined to find the new feature. The feature is used as input to the LR classifier model. Thus, the training process of GBDT + LR classifier is completed.

**FIGURE 5:**
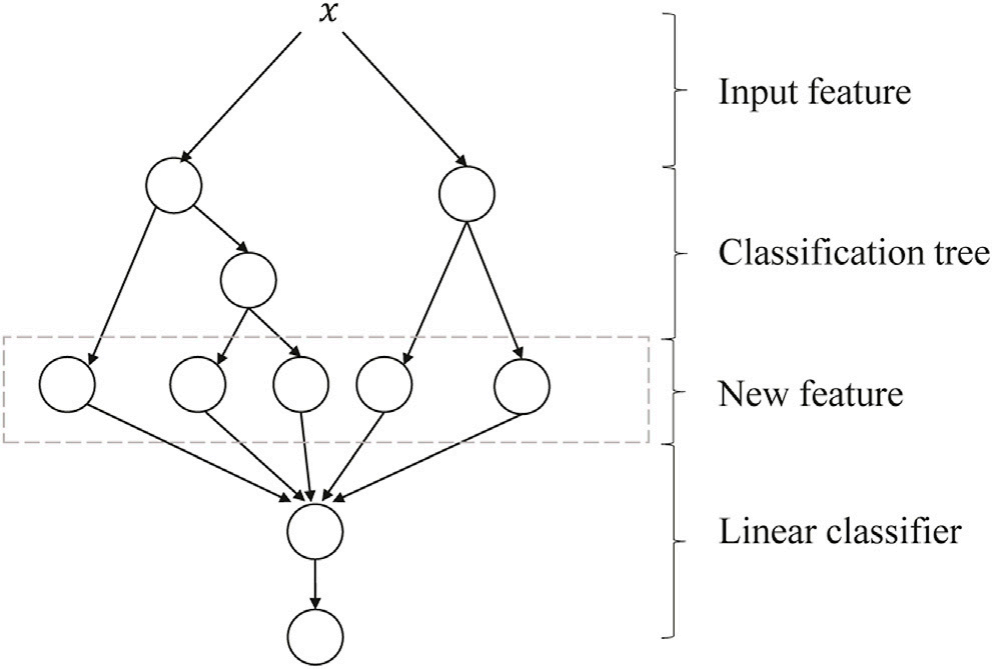
Algorithm structure of GBDT + LR. GBDT, Gradient Boosting Decision Tree; LR, logistic regression.

GBDT + LR is a process of feature crossing, and the path of GBDT can be directly used as the input feature of LR, avoiding the process of manual combination of cross features. Its algorithm structure is shown in Figure 5. The two trees in the figure are regression tree models trained by GBDT. The left tree has three leaf nodes, and the right tree has two leaf nodes. The final feature is a five-dimensional vector. For input *x*, it is assumed that it falls on the first node of the left tree and encodes (1, 0, 0); if it falls on the second node of the right tree, it encodes (0, 1), so the overall code is (1, 0, 0, 0, 1). Such codes are input into LR for classification. The steps of GBDT + LR for the algorithm are as follows:

**Step 1)** The original training data are trained with GDBT to generate a decision tree, and grid search is used to find the best parameter combination.

**A:** The initialization parameter of GDBT is shown in formula (22):

$$\Theta_{0}\left(x\right)=\frac{1}{2}*\log\left(\frac{\sum_{i=1}^{NUM}y_{i}}{\sum_{i=1}^{NUM}1-y_{i}}\right)$$ (22)

There are *NUM* samples to be trained, and *y*_*i*_ is the label for sample *i*. The loss function J (*y*, Θ_*t*_(*x*)) is defined as shown in formula (23):

$$J\left(y,\Theta_{t}\left(x\right)\right)=\log\left(1+\exp\left(-y\Theta_{t}\left(x\right)\right)\right)$$ (23)

where *y* is the label and Θ_*t*_(*x*) is the weak model in the *t*th round.

**B:** Cycle *t*, in turn, where *t* = 1, 2, …*T*.

1) The negative gradient of the loss function of sample *i*th in wheel *t*th is calculated, as follows:

$$r_{t,i}=-\frac{\partial J\left(y_{i},\Theta_{t-1}\left(x_{i}\right)\right)}{\partial \Theta_{t-1}\left(x_{i}\right)}=\frac{y_{i}}{\left(1+\exp\left(y_{i}\right)\Theta \left(x_{i}\right)\right)},i=1,2,\ldots ,NUM$$ (24)

where *i* = 1, 2, 3, …., *NUM*.

2) Construct the *t*th decision tree, and then get the corresponding leaf node area as *R*_*tn*_, where *n* = 1, 2, …, *N*. *N* is the number of leaf nodes of the tree.

3) For the samples in each leaf node, we calculated the *c*_*tn*_, which minimizes the loss function, as shown in formula (25):

$$c_{tn}={\mathrm{arg min}}_{c}\underset{x\in R_{tn}}{\sum }\log\left(1+\exp\left(-y_{i}\Theta \left(x_{i}\right)+c\right)\right)$$ (25)

4) Update the *t*th weak model as shown in formula (26):

$$\Theta_{t}\left(x\right)=\Theta_{t-1}\left(x\right)+\alpha *\overset{N}{\underset{n=1}{\sum }}c_{tn}I\left(x\in R_{tn}\right)$$ (26)

where *I* (*x* ∈ *R*_*tn*_) means that if *x* falls on a leaf node corresponding to *R*_*tn*_, then the corresponding term is 1, and *α* means the learning rate.

5) Determine whether *t* is greater than *T*. If *t* is less than *T*, *t* = *t* + 1 and jump to 1) for the next iteration. Otherwise, it means that all *T* weak learners have been constructed and jump to **C** to end the training.

**C:** The final strong learner model is shown in formula (27):

$$\Theta \left(x\right)=\Theta_{0}\left(x\right)+\alpha *\overset{T}{\underset{t=1}{\sum }}\overset{N}{\underset{n=1}{\sum }}c_{tn}I\left(x\in R_{tn}\right)$$ (27)

where *α* is the learning rate.

**Step 2)** After the training of GDBT, for each tree in the model, the calculated probability value of the leaf node is denoted as 1, and new training data are constructed. In this paper, One-Hot Encoding is used to process the results of GDBT and construct a new training dataset.

One-hot Encoding is also known as one-bit Efficient coding. Single-hot coding encodes N states by using an N-bit status register. Each of the N states has its own independent register bit, and only one is valid at any time such as the following.

General status Encoder: 000, 001, 010, 011, 100, 101

One-Hot Encoder: 000 001, 000 010, 000 100, 001 000, 010 000, 100 000

**Step 3)** The new features obtained and the label data of the original training data are input into the LR classifier for the training. The hypothesis function of LR is shown in formula (28). Given *x* and *θ*, the possibility that *x* belongs to a positive sample is shown in formula (29). *θ* is obtained by training to minimize the loss function in formula (30).

$$h_{\theta }\left(x\right)=g\left(\theta^{T}x\right),g\left(z\right)=\frac{1}{1+e^{-z}}$$ (28)

$$\mathrm{Pr}\left(y=1|x;\theta \right)=h_{\theta }\left(x\right)=\frac{1}{1+e^{-\theta^{T}x}}$$ (29)

$$L\left(\theta \right)=-\frac{1}{m}\left[\overset{m}{\underset{i=1}{\sum }}y^{\left(i\right)}\log h_{\theta }\left(x^{\left(i\right)}\right)+\left(1-y^{\left(i\right)}\right)\log\left(1-h_{\theta }\left(x^{\left(i\right)}\right)\right)\right]$$ (30)

## 3 Result and Discussion

### 3.1 Dataset

The data are downloaded from the lncRNA–disease-associated data from the lncRNADisease database, including the data of three versions, namely, the version of June 2012, the version of January 2014, and the version of June 2015, labeled as DS1, DS2, and DS3, respectively. The training samples are obtained by all positive samples and randomly selecting negative samples by K-means clustering.

### 3.2 Performance Measures

In this paper, the 10-fold cross-validation is selected to measure the performance of the proposed method. The parameters of GBDTLRL2D are shown in Table 2. The main steps of 10-fold cross-validation are as follows: the training set is randomly divided into 10 subsets of the same size, nine of which are used as training data, and the remaining one is used as validation data in each training. After ten times of the above process training, each of the ten subsets, in turn, is used as validation data to obtain ten performance results. The final performance evaluation is obtained by averaging the ten performance results. Various evaluation indexes are used in this experiment, including Recall (REC), F1-score, Accuracy (ACC), Matthews correlation coefficient (MCC), and area under the receiver operating characteristic (ROC) curve (AUC). Their definition is as follows:

$$\text{ Recall }=\frac{TP}{TP+FN}$$ (31)

$$\text{ Accuracy }=\frac{TP+TN}{TP+TN+FP+FN}$$ (32)

$$F1_{\text{score }}=\frac{2*TP}{2TP+FP+FN}$$ (33)

$$\mathrm{MCC}=\frac{TP*TN-FP*FN}{\sqrt{\left(TP+FP\right)\left(TP+FN\right)\left(TN+FP\right)\left(TN+FN\right)}}$$ (34)

where TP represents the number of correct prediction of positive samples as positive samples, TN represents the number of correct prediction of negative samples as negative samples, FN represents the number of incorrect prediction of positive samples as negative samples, and FP represents the number of prediction of negative samples as positive samples. We plot the ROC based on the true positive rate (TPR) and false positive rate (FPR), and we calculate the AUC as an important index to measure the model.

**TABLE 2:** The partial experimental parameters of GBDTLRL2D.

Notation	Value	Definition
*nl*_1_	112	The number of lncRNAs in dataset1
*nd*_1_	150	The number of diseases in dataset1
*n*_1_	262	Total number of diseases and lncRNAs in dataset1
*nl*_2_	131	The number of lncRNAs in dataset2
*nd*_2_	169	The number of diseases in dataset2
*n*_2_	300	Total number of diseases and lncRNAs in dataset2
*nl*_3_	285	The number of lncRNAs in dataset3
*nd*_3_	226	The number of diseases in dataset3
*n*_3_	511	Total number of diseases and lncRNAs in dataset3
$\gamma_{l}^{\prime }$	1	Gaussian interaction properties of lncRNA kernel similar bandwidth
$\gamma_{d}^{\prime }$	1	Gaussian interaction properties of lncRNA kernel similar bandwidth
*k*	10	K-means clustering divides the unknown samples into k clusters
*K*	5	The number of negative samples taken in MetaGraph2Vec

Note. lncRNA, long noncoding RNA.

### 3.3 Performance Comparison With Existing Machine Learning Methods

**FIGURE 6:**
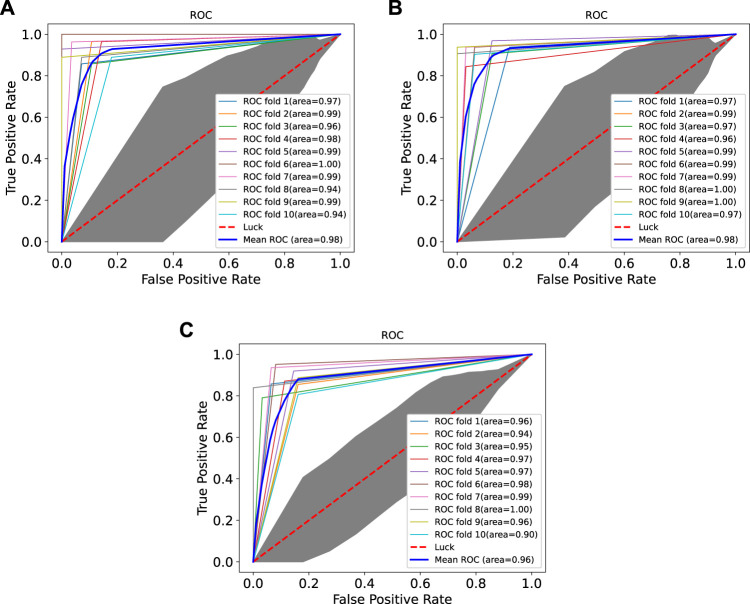
ROC curve of GBDTLRL2D on three datasets. ROC, receiver operating characteristic.

**FIGURE 7:**
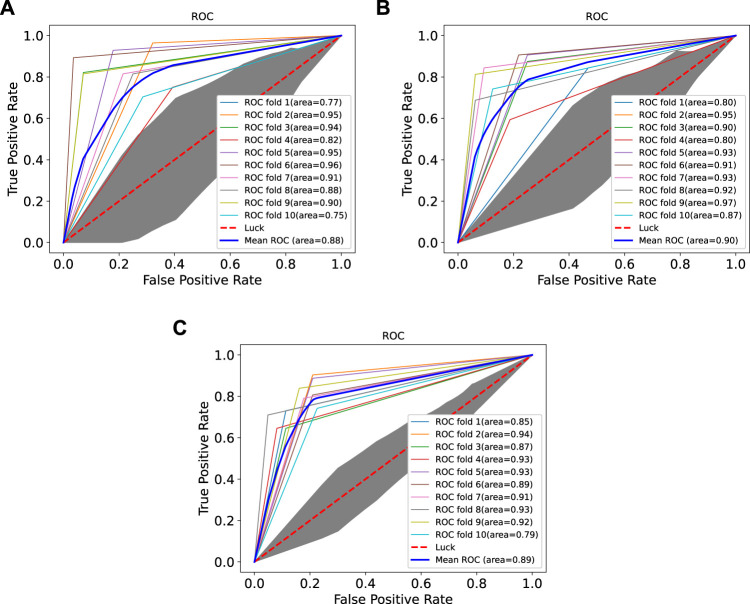
ROC curves on three datasets using RF + LR. ROC, receiver operating characteristic; RF, random forest; LR, logistic regression.

**FIGURE 8:**
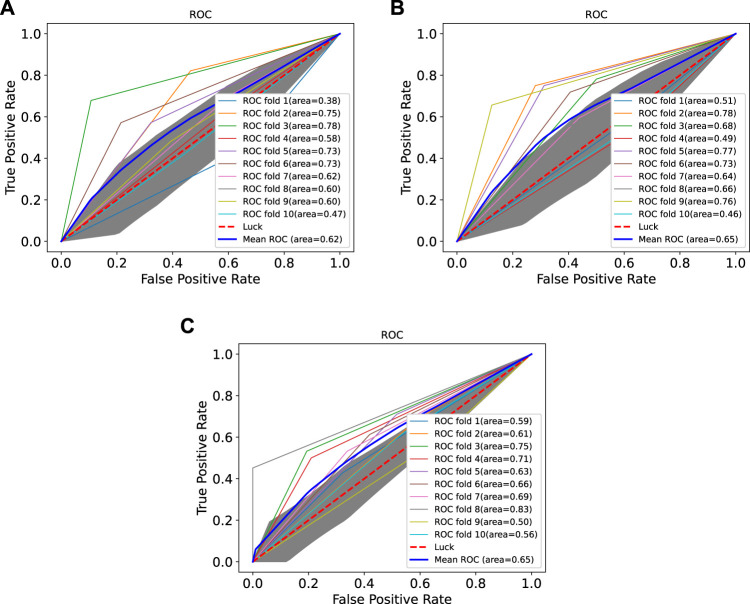
ROC curves on three datasets using GBDT. ROC, receiver operating characteristic.

**FIGURE 9:**
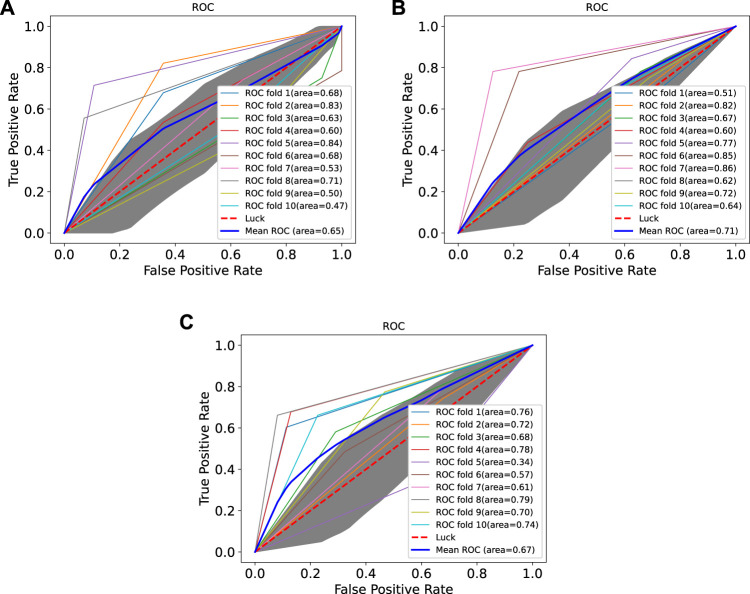
ROC curves on three datasets using LR. ROC, receiver operating characteristic; LR, logistic regression.

In order to prove the advantages of GBDT combined with LR classifier, we carried out several experiments to compare with GBDTLRL2D, including using RF + LR as the classifier, using GBDT only as the classifier, and using LR only as a classifier. It can be seen that GBDTLRL2D obtains the best performance among these methods. The 10-fold cross-validation is selected to measure the performance of the proposed method. Table 3 shows the predictive performance of GBDT + LR compared with other methods. The ROC curves of 10-fold cross-validation of GBDTLRL2D, RF + LR, GBDT, and LR are shown in Figures 6–9, respectively. The AUCs of GBDTLRL2D, RF + LR, GDBBT, and LR in DS1 are 0.976, 0.880, 0.619, and 0.649, respectively. The AUCs of GBDTLRL2D, RF + LR, GDBBT, and LR in DS2 are 0.983, 0.898, 0.654, and 0.705, respectively. The AUCs of GBDTLRL2D, RF + LR, GDBBT, and LR in DS3 are 0.961, 0.889, 0.647, and 0.667, respectively. It can be seen that GBDTLRL2D obtains the best performance among these methods.

**TABLE 3:** Comparison of prediction performance using other machine learning methods.

Dataset	Method	ACC	Recall	*F*1_*score*_	MCC	AUC
DS1	GBDT + LR	0.928	0.920	0.927	0.858	0.975
DS2		0.934	0.928	0.934	0.870	0.982
DS3		0.887	0.871	0.885	0.777	0.961
DS1	RF + LR	0.787	0.767	0.780	0.581	0.880
DS2		0.800	0.802	0.801	0.603	0.898
DS3		0.796	0.767	0.790	0.601	0.889
DS1	GBDT	0.570	0.658	0.608	0.125	0.619
DS2		0.600	0.724	0.645	0.210	0.654
DS3		0.636	0.631	0.636	0.282	0.647
DS1	LR	0.570	0.659	0.609	0.125	0.649
DS2		0.601	0.724	0.645	0.211	0.705
DS3		0.636	0.631	0.636	0.282	0.667

Note. lncRNA, long noncoding RNA; ACC, Accuracy; MCC, Matthews correlation coefficient; AUC, area under the receiver operating characteristic curve; GBDT, Gradient Boosting Decision Tree; LR, logistic regression; RF, random forest.

### 3.4 Performance Comparison With Different Topological Features

In order to demonstrate the performance of the experimental features, different feature groups (not using MetaGraph2Vec for representation learning, but using MetaGraph2Vec for representation learning) and different negative samples (not using K-means for clustering, but using K-means for clustering) are used for performance comparison in this section. Table 4 and Table 5 show the performance comparison with different topological features. In Table 4, on the same dataset, the result shows that the features obtained through MetaGraph2Vec embedding learning are trained to achieve better performance. Similarly, in Table 5, the performance of negative samples obtained through K-means cluster screening is better than that of negative samples randomly selected for training.

**TABLE 4:** Performance comparison of representation learning without MetaGraph2vec.

Dataset	Whether to use MetaGraph2Vec	ACC	Recall	*F*1_*score*_	MCC	AUC
DS1	Yes	0.928	0.920	0.927	0.858	0.975
DS2		0.934	0.928	0.934	0.870	0.982
DS3		0.887	0.871	0.885	0.777	0.961
DS1	No	0.773	0.785	0.779	0.555	0.871
DS2		0.786	0.758	0.778	0.581	0.877
DS3		0.829	0.826	0.829	0.667	0.923

Note. lncRNA, long noncoding RNA; ACC, Accuracy; MCC, Matthews correlation coefficient; AUC, area under the receiver operating characteristic curve.

**TABLE 5:** Performance comparison without K-means.

Dataset	Whether to use K-means	ACC	Recall	*F*1_*score*_	MCC	AUC
DS1	Yes	0.928	0.920	0.927	0.858	0.975
DS2		0.934	0.928	0.934	0.870	0.982
DS3		0.887	0.871	0.885	0.777	0.961
DS1	No	0.802	0.713	0.778	0.617	0.888
DS2		0.769	0.705	0.745	0.553	0.871
DS3		0.779	0.726	0.763	0.562	0.876

Note. ACC, Accuracy; MCC, Matthews correlation coefficient; AUC, area under the receiver operating characteristic curve.

### 3.5 Performance Comparison With Existing Methods

To further illustrate the advantages of the proposed model, several existing methods based on embedding are compared with GBDTLRL2D, such as LDAH2V, VGAELDA (Shi et al., 2021), and GCNMDA (Long et al., 2020). The 10-fold cross-validation is selected to measure the performance.

LDAH2V: The LDAH2V uses the HIN2Vec to calculate the meta-path and feature vector for each lncRNA–disease pair in the heterogeneous information network (HIN), which consists of lncRNA similarity network, disease similarity network, miRNA similarity network, and the associations between them. Then, a Gradient Boosting Tree (GBT) classifier to predict lncRNA–disease associations is built with the feature vectors.
VGAELDA: The VGAELDA integrates graph embedding learning and the alternate training via variational inference. Variational graph autoencoders (VGAEs) infer representations from features of lncRNAs and diseases, while graph autoencoders propagate labels via known lncRNA–disease associations. These two kinds of autoencoders are trained alternately by adopting variational expectation–maximization algorithm.
GCNMDA: The graph convolution network is used for network embedding in GCNMDA. The GCNMDA exploited the Conditional Random Field (CRF), which can ensure that similar nodes have similar representations. At the same time, the attention mechanism is designed in CRF layer.

**FIGURE 10:**
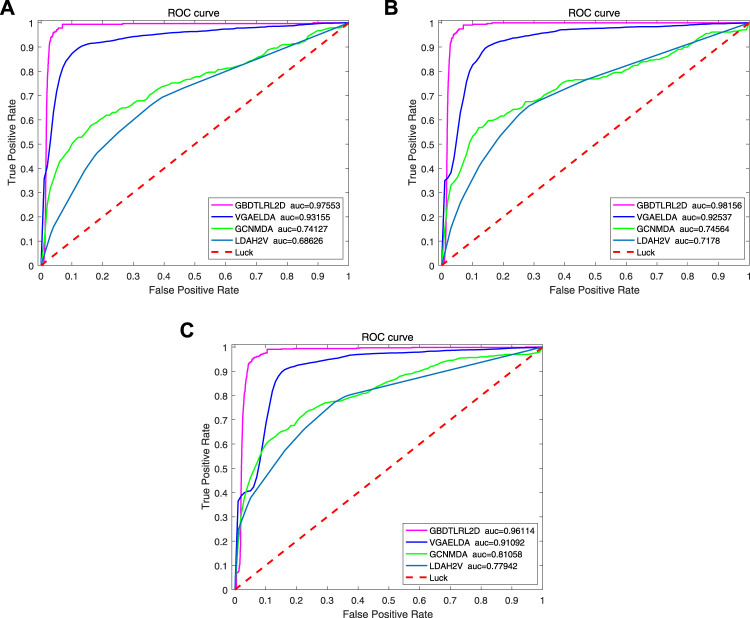
ROC curve comparison with existing methods. ROC, receiver operating characteristic.

Figure 10 shows the comparison results. Among these methods, the proposed model GBDTLRL2D achieves the best performance. There are several reasons: 1) the features learned by MetaGraph2Vec can better preserve node information and semantic information in a heterogeneous information network. 2) K-means clustering is used to select more representative negative samples. 3) The GBDTLRL2D uses the combined machine learning method of GBDT + LR with good performance to make predictions.

Despite that our method is obviously superior to previous methods in all aspects, there are some limitations to GBDTLRL2D. The number of lncRNA–disease associations confirmed by biological experimental methods is limited. In addition, it is important to select classifiers. Currently, GBDT + LR is the best classifier for our model. In the future, we will have to try to combine other classifiers to achieve more accurate predictions.

### 3.6 Case Study

In this section, to further show the performance of the proposed model GBDTLRL2D in predicting the lncRNA–disease association, a case study is conducted on lncRNA “PVT1.” A proven association between “PVT1” and many diseases has been found in biology. In this paper, the proposed model GBDTLRL2D is used to predict the association between “PVT1” and disease. After processing by our algorithm, the list of diseases associated with lncRNA “PVT1” and their predicted scores is obtained. Ranking the diseases according to the predicted score from large to small, we can find that all the diseases in the top 10 associated with lncRNA “PVT1” are confirmed to be associated with “PVT1” in the lncRNADisease database. The top 10 diseases associated with lncRNA “PVT1” and their predicted scores are shown in Table 6.

**TABLE 6:** The top 10 predicted diseases related to “PVT1.”

Rank	Disease	Score
1	Lymphoma	0.999 169 371
2	Cancer	0.998 948 531
3	Breast cancer	0.998 948 531
4	Prostate cancer	0.998 948 531
5	Ovarian cancer	0.998 948 531
6	Type 2 diabetes	0.995 265 292
7	Type 1 diabetes	0.995 265 292
8	Diabetic nephropathy	0.987 846 199
9	Hodgkin’s lymphoma	0.984 907 726
10	Burkitt’s lymphomas	0.983 042 458

## Conclusions

LncRNAs have been found by biologists to be closely related to diseases. Predicting the lncRNA–disease associations is conducive to research on the pathogenesis of a disease. But traditional biological methods have a large amount of data and are expensive, labor-intensive, and time-consuming. In recent years, there has been much research on computational models of biological experiments. In this paper, a method for predicting lncRNA–disease association is proposed. The proposed method uses MetaGraph2Vec to learn the features of nodes in a heterogeneous network and then uses K-means to select representative negative samples to solve the problem of imbalance between positive and negative samples, and the GBDT combined with LR is used as a classifier to predict lncRNA–disease associations. At last, the average AUCs of GBDTLRL2D obtained on the three datasets are 0.98, 0.98, and 0.96 in 10-fold cross-validation. Compared with the SIMCLDA, IIRWR, NCPLDA, and other experiments, the GBDTLRL2D greatly improves accuracy and performance.

## Data Availability

Publicly available datasets were analyzed in this study. These data can be found here: http://www.cuilab.cn/lncrnadisease.
